# Efficacy of extracorporeal shock wave therapy combined with conventional physical therapy for chronic low back pain: a systematic review and network meta-analysis of randomized controlled trials

**DOI:** 10.3389/fphys.2026.1807929

**Published:** 2026-06-10

**Authors:** Zhengtong Qiao, Shizhen Xi, Kai Yang, Feng Gu, Xin Zhao, Pan Hou, Yapeng Li, Yanan Feng, Zhijie Zhang

**Affiliations:** 1 Rehabilitation Therapy Center, Luoyang Orthopedic-Traumatological Hospital, Henan Provincial Orthopedic Hospital, Luoyang, China; 2 Knee Surgery Center, Luoyang Orthopedic-Traumatological Hospital, Henan Provincial Orthopedic Hospital, Luoyang, China

**Keywords:** chronic low back pain, extracorporeal shock wave therapy, network meta-analysis, non-invasive intervention, physical therapy

## Abstract

**Objectives:**

To compare the clinical effects of extracorporeal shock wave therapy (ESWT) combined with conventional physical therapy (CPT) versus monotherapy for chronic low back pain (CLBP).

**Methods:**

A frequentist network meta-analysis was conducted in Stata/MP 18 using the *network* package. PubMed, Web of Science, Embase, and the Cochrane Library were searched from inception to April 11, 2026. Randomized controlled trials (RCTs) evaluating ESWT with or without CPT in adults with CLBP were included. Two reviewers independently screened studies; disagreements were resolved by consensus with a third reviewer. Two reviewers independently extracted study characteristics, intervention details, and outcomes. Risk of bias was assessed using RoB 2.0, and certainty of evidence using GRADE. Treatment ranking probabilities were estimated using surface under the cumulative ranking curves (SUCRA).

**Results:**

Fourteen RCTs reported pain score (VAS/NRS) and eight reported functional score (ODI); lower scores indicate improvement. According to SUCRA ranking, combined therapy ranked first for both pain relief (96.0%) and functional improvement (99.3%), followed by ESWT, CPT, and sham ESWT in sequence. Combined therapy (ESWT with CPT) provided greater pain relief than sham ESWT (SMD = −1.70; 95% CI: −2.69 to −0.71) and CPT alone (SMD = −0.88; 95% CI: −1.29 to −0.47). ESWT was also superior to sham ESWT (SMD = −1.22; 95% CI: −1.83 to −0.61). For functional improvement, combined therapy achieved significantly greater reductions in ODI than ESWT (MD = -3.60; 95% CI -6.70 to -0.51), sham ESWT (MD = −7.60; 95% CI: −14.00 to −1.12), and CPT (MD = −5.29; 95% CI: −7.53 to −3.06).

**Conclusion:**

Combined therapy shows promise for alleviating pain and improving function in patients with CLBP. ESWT may help reduce pain, but no significant effects on functional outcomes have been observed. However, more high-quality randomized controlled trials are needed to confirm these findings.

**Systematic review registration:**

https://www.crd.york.ac.uk/prospero/, identifier 420251170304.

## 1 Introduction

Chronic low back pain (CLBP) is a clinical syndrome characterized by persistent low back pain lasting for 12 weeks or more (Nicol et al., 2023; Zheng et al., 2025). It can be broadly categorized into myofascial, discogenic, and facet joint-related subtypes (Zhai et al., 2024; Zheng et al., 2025). Epidemiological data indicates that approximately 619 million individuals worldwide were affected by low back pain in 2020, with projections suggesting this number will increase to 843 million by 2050 (Baran et al., 2022; GBD 2021 Low Back Pain Collaborators, 2023). The long-term pain associated with CLBP significantly impairs patients’ quality of life, making it a pressing global public health issue (Baran et al., 2022; GBD 2021 Low Back Pain Collaborators, 2023; Tieppo et al., 2024).

Extracorporeal shock wave therapy (ESWT), a non-invasive modality, has gained attention in CLBP management for its remarkable analgesic efficacy (Simplicio et al., 2020; Yue et al., 2021; Wang et al., 2024). The mechanism of ESWT involves the high-energy pressure wave to target tissues, inducing mechanical transduction effects that provide analgesia, modulate inflammation, promote angiogenesis, improve local microcirculation, and facilitate tissue repair (Tenforde et al., 2022; Zhang and Ma, 2023). However, studies have indicated that ESWT yields no significant long-term functional improvements in CLBP patients (Rajfur et al., 2022; Tan et al., 2024).

Conventional physical therapy (CPT) has long been regarded as one of the preferred interventions for the CLBP clinical management (Hayden et al., 2021; Rizzo et al., 2025). However, Hayden et al. noted that although CPT consistently improves long-term functional outcomes in CLBP management, the quality of evidence supporting its efficacy in short-term pain relief is relatively low (O’Keeffe et al., 2020; Hayden et al., 2021). Meanwhile, Gilanyi et al. pointed out that adherence to conventional rehabilitation regimens is poor, particularly in populations presenting with significant pain symptoms (Gilanyi et al., 2024; Nevelikova et al., 2025).

Accumulating evidence from clinical studies has supported that the combination of ESWT and CPT confers distinct advantages in pain alleviation and functional recovery (Liu et al., 2023; Cashin et al., 2025; Zhang et al., 2025). However, ESWT treatment protocols vary substantially across studies, and the efficacy of the combined therapy remains inconclusive (Elgendy et al., 2020; Burton, 2022). The present study employed a network meta-analysis (NMA) to explore the efficacy of combined therapy and provide evidence-based insights to guide its rational clinical application.

## 2 Methods

This NMA was reported in accordance with the relevant extension of the Preferred Reporting Items for Systematic Reviews and Meta-analyses (PRISMA) reporting guideline (Page et al., 2021). This review was registered in PROSPERO (420251170304).

### 2.1 Search strategy

Four electronic databases, including PubMed, Web of Science, Embase, and the Cochrane library, were searched from inception to April 11, 2026. A supplementary search of the ClinicalTrials.gov trial registry was conducted to identify ongoing or unpublished studies. Search strategies combined free-text terms and controlled vocabulary where applicable for “extracorporeal shock wave therapy” and “low back pain.” In addition, the reference lists of included studies and relevant review articles were screened to identify potentially eligible studies. The detailed search strategies for each database are provided in **Supplementary Material A**.

### 2.2 Literature screening

Citations retrieved from databases were imported into EndNote version 20 (Clarivate Analytics), and duplicate citations were removed. Two authors independently reviewed the titles and abstracts of each record. The full text was retrieved for potentially eligible studies, and the same 2 authors independently reviewed the full texts, selected studies that met the eligibility criteria for inclusion, and conducted data extraction. Any disagreements were resolved by discussion or by consultation with other authors.

### 2.3 Inclusion and exclusion criteria

We identified eligible studies according to PICOS. Population: adults with CLBP (pain duration >3 months), including chronic nonspecific low back pain and closely related lumbopelvic pain syndromes reported as low back pain in the original trials. Intervention: ESWT alone or ESWT combined with CPT. Comparator: sham ESWT or CPT alone. Outcomes: pain intensity (VAS/NRS) and disability/function (ODI). Study design: RCTs. Sham ESWT was defined as zero-energy output or the use of an energy-blocking transmission medium. CPT included stretching, strengthening, and other routine rehabilitation modalities. When trials were multi-arm studies or included additional intervention arms outside the predefined network nodes (e.g., injections or pharmacological treatments), only arms that could be mapped to ESWT, sham ESWT, CPT, or combined therapy were included in the network, and non-mappable arms were excluded.

### 2.4 Data extraction

Two reviewers independently extracted the primary information from a pre-set standardized form. The following data were extracted (1): Basic information, including first author and year of publication. (2) Characteristics of participants, including sample size, age, and gender. (3) Intervention details include the treatment, dosage, duration, process, and follow-up period. (4) Outcomes information. When multiple follow-up time points were reported, the earliest post-intervention or nearest common follow-up time point across studies was used for quantitative synthesis. If the data were ambiguous, the authors of the individual studies were contacted by email or the data were obtained using the image−recognition software GetData v2.26.

### 2.5 Risk of bias within individual studies

Two reviewers independently assessed risk of bias using the Cochrane Risk of Bias tool, version 2.0 (RoB 2.0) (Sterne et al., 2019); disagreements were resolved by a third reviewer. RoB 2.0 evaluates five domains: bias arising from the randomization process; bias due to deviations from intended interventions; bias due to missing outcome data; bias in measurement of the outcome; and bias in selection of the reported result. For each study, domain-level judgments were low risk, some concerns, or high risk. Overall judgments followed RoB 2.0 guidance: low risk if all domains were low risk; some concerns if ≥1 domain had some concerns and none were high risk (also including cases with insufficient information or non-applicable items); and high risk if any domain was high risk.

### 2.6 Certainty of evidence

We assessed certainty of evidence using GRADE (Guyatt et al., 2011; Brozek et al., 2021; Qiao et al., 2025). Because the evidence base comprised randomized trials, comparisons started at high certainty and could be downgraded for risk of bias, inconsistency (including heterogeneity), indirectness (including potential intransitivity in networks), imprecision, and publication bias. Publication bias was examined using funnel plots; for network meta-analysis, comparison-adjusted funnel plots were used. For the network meta-analysis, we first graded direct estimates. We then graded indirect estimates by taking the lower rating of the two contributing direct comparisons in the dominant first-order loop and considering transitivity. For each network estimate, we adopted the higher of the direct and indirect ratings and then evaluated incoherence (network inconsistency) and imprecision to determine the final certainty rating.

### 2.7 Statistical analysis

The main characteristics of the included studies were qualitatively summarized. A frequentist network meta-analysis was conducted in Stata/MP 18 using the *network* package, whereas pairwise meta-analyses were performed using standard Stata commands. We evaluated clinical similarity and the transitivity assumption; statistical consistency was assessed using node-splitting (local) and the design-by-treatment interaction model (global). Network geometry was displayed as a four-node plot (ESWT, Sham ESWT, CPT, and Combined therapy) for each outcome; node size and edge thickness were proportional to the number of participants per intervention and the number of direct-comparison trials, respectively. Pain outcomes were summarized as standardized mean differences (SMD) with 95% confidence intervals (CIs), and function outcomes as mean differences (MD) with 95% CIs. Effects were coded so that negative SMDs/MDs favored the active intervention (greater improvement). Small-study effects (publication bias) were explored using funnel plots. Treatment ranking was summarized using the surface under the cumulative ranking curve (SUCRA; 0–100%, higher values indicating more effective interventions). Due to the limited number of included studies, sensitivity analyses were performed to assess the robustness of the study findings. All tests were two-sided with *P* < 0.05 considered statistically significant.

## 3 Results

### 3.1 Literature screening process and results

**Figure 1:**
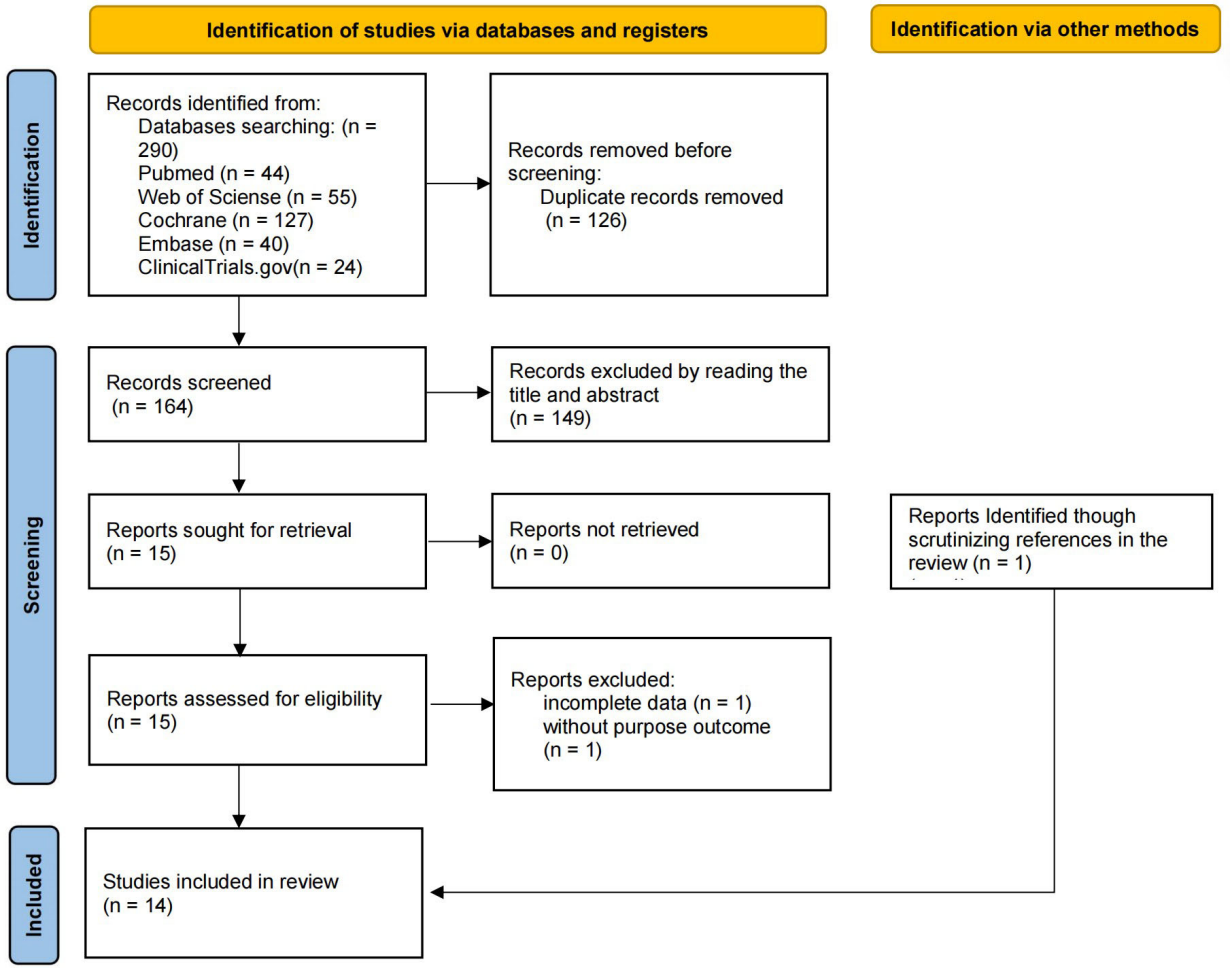
Flow diagram of the studies screened and included according to the PRISMA.

The PRISMA flowchart was presented in **Figure 1** (Page et al., 2021). The initial electronic search identified 290 potentially relevant publications. After removing 126 duplicate records, a total of 164 records were screened based on reading titles and abstracts, resulting in the exclusion of 149 records. Among the remaining 15 studies eligible for full-text review, 2 were excluded based on inclusion and exclusion criteria (Schneider, 2018; Kong et al., 2023). Additionally, one eligible study was retrieved by manually screening the reference lists. Ultimately, 14 RCTs (n = 738) were included.

### 3.2 Description of included studies

**Figure 2:**
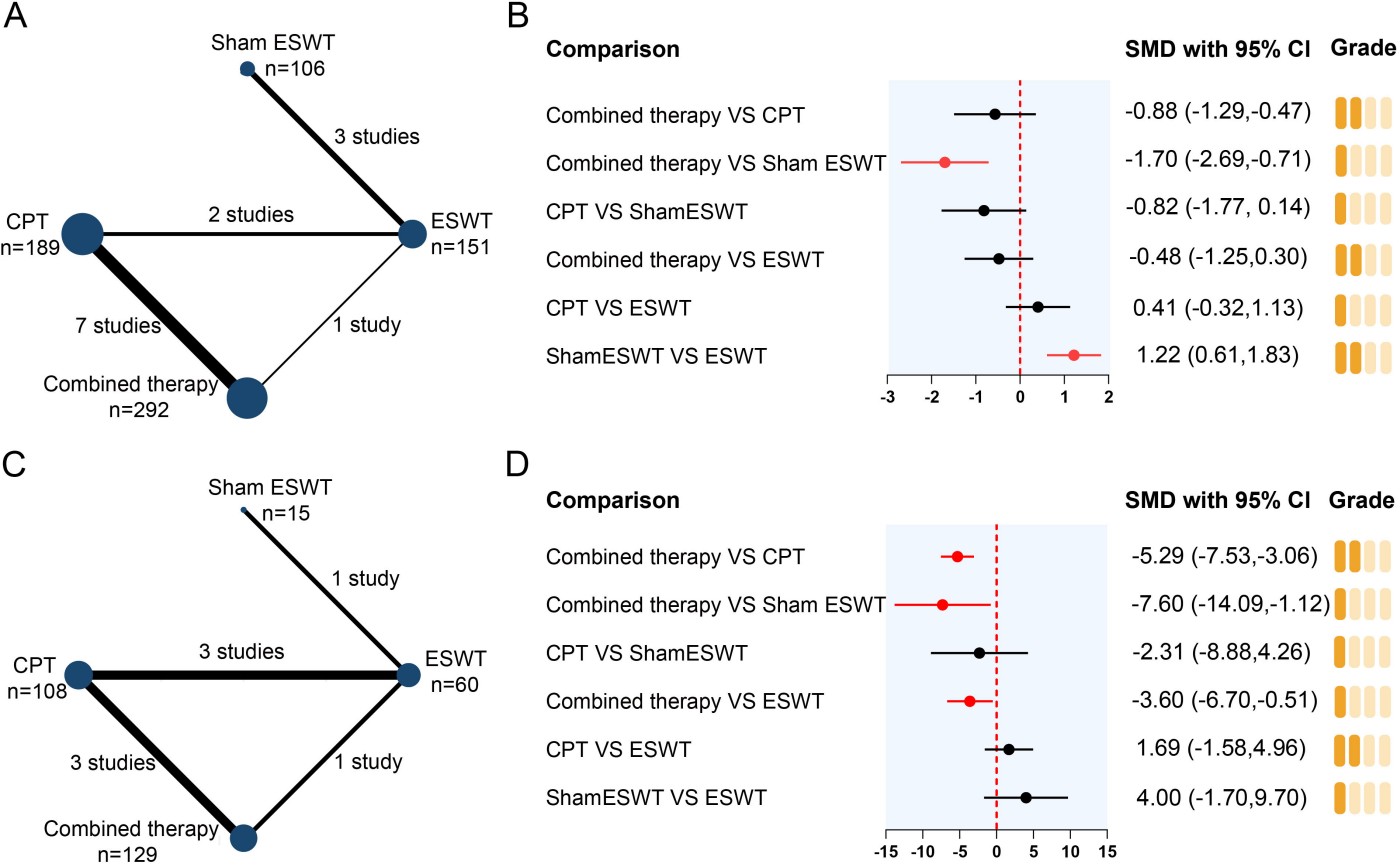
Network Maps and Interval Plots. Interval plot and forest of pain **(A, B)**, Interval plot and forest of function **(C, D)**. Node size and edge thickness were proportional to the number of participants per intervention and the number of direct-comparison trials, respectively. Red confidence interval lines indicate a statistically significant difference. For GRADE assessment, two dark yellow blocks represent low certainty of evidence, and one dark yellow block represents very low certainty of evidence. ESWT, extracorporeal shock wave therapy; Sham ESWT, sham extracorporeal shock wave therapy; CPT, Conventional physical therapy; Combined therapy, ESWT with conventional physical therapy; SMD, standardized mean difference; MD, mean difference; CI, confidence interval.

The meta-analysis included 14 studies (Lee et al., 2014; Han et al., 2015; Moon et al., 2017; Walewicz et al., 2019; Eftekharsadat et al., 2020; Elgendy et al., 2020; Guo et al., 2021; Kızıltaş et al., 2022; Rajfur et al., 2022; Wu et al., 2023; Back et al., 2024; Tan et al., 2024; Fu et al., 2026; Nedelka et al., 2025) with 738 participants (**Table 1**). A total of 8 countries contributed to the publication of these studies, with China being the most prolific (4 studies, 28.6%). The average age of the participants was 47.5 years. The intervention duration ranged from a single session to 6 weeks, with an intensity of 0.0298-0.35 mJ/mm². Among these interventions, ESWT was included in five studies, Sham ESWT in three studies, CPT in eight studies, and Combined therapy in ten studies. Fourteen studies included pain score as an outcome measure and eight studies reported ODI results (**Figure 2A**; **Supplementary Figures 1**, **2**). The specific ESWT parameters can be found in **Supplementary Materials** (**Supplementary Table 1**).

**Table 1:** Summary of studies characteristics.

Author, year	Country	Disease type	Intervention	Mean (SD) age, y	Gender M/F	Duration	Frequency	Outcomemeasures
Elgendy et al., 2020	Egypt	Non-specific chronic low back pain	Combined therapy CPT	32.73(6.73) 33.26(5.48)	5/10 5/10	6 weeks	twice a week	VAS
Han et al., 2015	Korea	chronic low back pain	ESWT CPT	49.7(8.3) 46.0(8.9)	15 15	6 weeks.	twice a week	VAS; ODI
Guo et al., 2021	China	nonspecific low back pain	Combined therapy	34.9 (8.7)	25/22	4 weeks.	twice a week	NRS
Tan et al., 2024	China	postpartum patients with sacroiliac joint dysfunction	ESWT CPT Combined therapy	30.73(0.72) 30.56(0.73) 30.33(0.74)	30 30 30	2 weeks	twice a week	VAS; ODI
Kızıltaş et al., 2022	Türkiye	chronic low back pain	Combined therapy CPT	47.4(14.3) 45.3(12.2)	23/13 14/20	1 week	twice a week	VAS; ODI
Lee et al., 2014	Korea	Chronic Low Back Pain	Combined therapy CPT	53.92(10.38) 54.33(13.16)	13 15	6 weeks	twice a week	VAS
Rajfur et al., 2022	Poland	Chronic Low Back Pain	Combined therapy CPT	43.0(13.1) 45.4(14)	10/10 10/10	5 weeks	twice a week	VAS; ODI
Moon et al., 2017	Korea	sacroiliac joint pain	ESWT Sham ESWT	54.42(19.05) 59.18(15.30)	3/11 1/10	single session	NR	VAS; ODI
Walewicz et al., 2019	Poland	Chronic Low Back Pain	Combined therapy CPT	51.1(8.4) 55.8(9.3)	6/14 5/15	6 weeks	twice a week	VAS; ODI
Eftekharsadat et al., 2020	Iran	Low Back Pain	ESWT	44.74(9.34)	7/20	5 weeks	once a week	VAS
Back et al., 2024	Brazil	chronic non-specific low back pain	ESWT Convex tips Sham ESWT	56(11.6) 51(12.2) 48(1.5)	10/18 6/21 8/19	single session	NR	NRS
Fu et al., 2026	China	piriformis syndrome	Combined therapy	60.4(12.7)	12/23	single session	NR	VAS; ODI
Wu et al., 2023	China	low back pain	ESWT Thermomagnetic	51.37(9.39) 52.62(9.91)	26/25 31/25	4 sessions	Once every 4–5 days	VAS
Nedelka et al., 2025	Czech Republic	lumbar facet joint pain	ESWT Sham ESWT	44 39	33/31 35/29	5 weeks	once a week	VAS

ESWT indicates extracorporeal shock wave therapy; Sham ESWT, sham extracorporeal shock wave therapy; CPT, Conventional physical therapy; Combined therapy, ESWT with conventional physical therapy; VAS, Visual Analogue Scale; NRS, Numeric Rating Scale; ODI, Oswestry Disability Index.

### 3.3 Network effect estimates

#### 3.3.1 Pain outcome

In the network meta-analysis for pain (negative SMD indicates greater pain reduction), ESWT reduced pain compared with sham ESWT (SMD −1.22; 95% CI −1.83 to −0.61). In contrast, Combined therapy (SMD −0.48; 95% CI −1.25 to 0.30) and CPT (SMD 0.41; 95% CI −0.32 to 1.13) did not differ significantly from ESWT. Compared with Sham ESWT, CPT showed no significant difference (SMD −0.82; 95% CI −1.77 to 0.14), whereas Combined therapy showed significantly greater pain improvement (SMD −1.70; 95% CI −2.69 to −0.71). Compared with CPT, Combined therapy also produced significantly greater pain improvement (SMD −0.88; 95% CI −1.29 to −0.47) (**Figures 2A, C**; **Supplementary Figure 3**).

#### 3.3.2 Functional outcome

For ODI (lower scores indicate better function), the network estimates suggested no significant differences between ESWT and Sham ESWT (MD −4.00; 95% CI −9.70 to 1.70) or CPT (MD −1.69; 95% CI −4.96 to 1.58). CPT did not differ significantly from Sham ESWT (MD −2.31; 95% CI −8.88 to 4.26). In contrast, Combined therapy showed significant advantages over ESWT (MD -3.60; 95% CI -6.70 to -0.51), Sham ESWT (MD -7.60; 95% CI -14.09 to -1.12) and over CPT (MD -5.29; 95% CI -7.53 to -3.06) (**Figures 2B, D**; **Supplementary Figure 4**).

### 3.4 Pairwise meta-analyses

**Figure 3:**
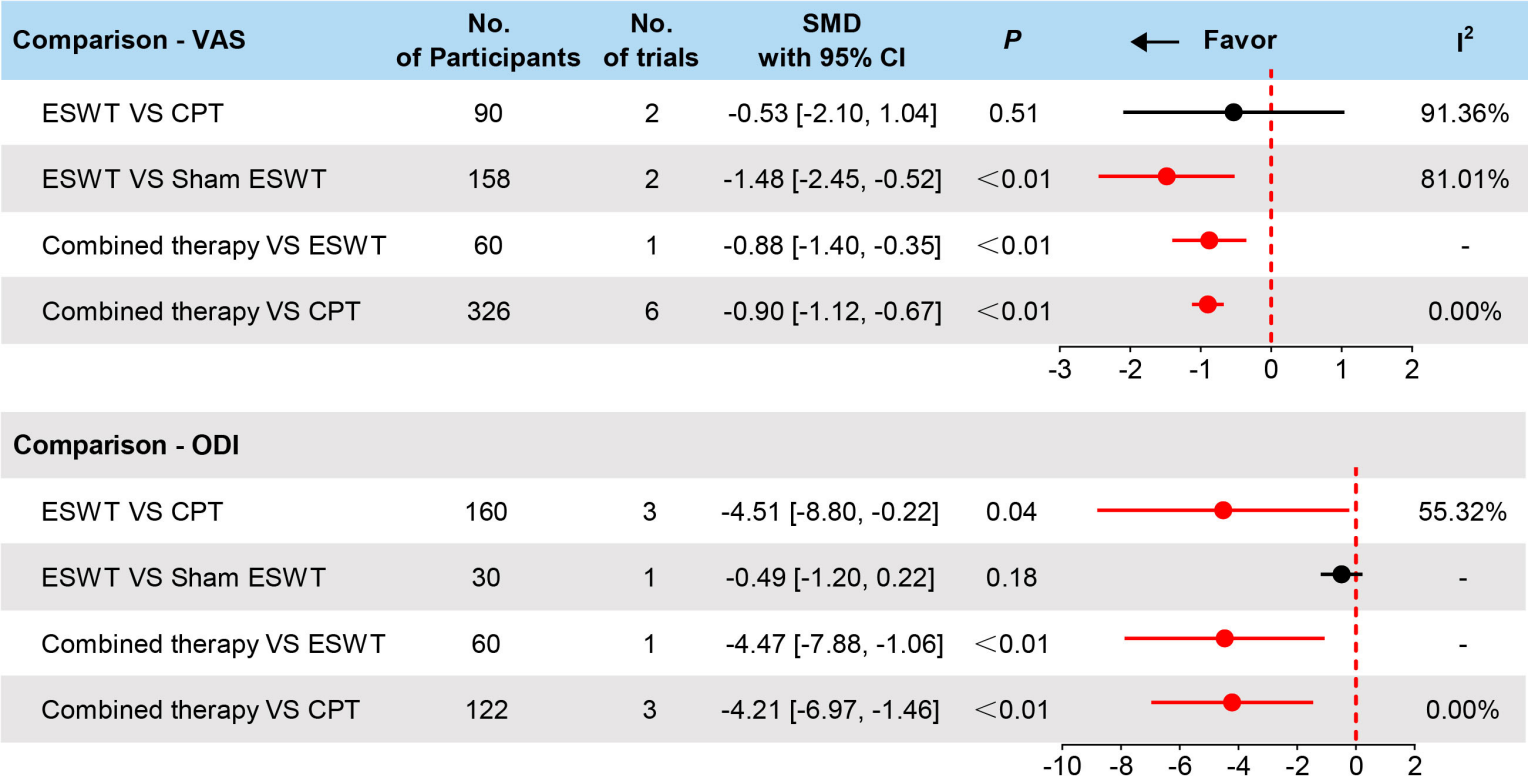
Pairwise forest plots. VAS, Visual Analogue Scale; ODI, Oswestry Disability Index; ESWT indicates extracorporeal shock wave therapy; Sham ESWT, sham extracorporeal shock wave therapy; CPT, Conventional physical therapy; Combined therapy, ESWT with conventional physical therapy; SMD, standardized mean difference; CI, confidence interval.

For VAS, ESWT did not differ significantly from CPT (MD −0.53; 95% CI −2.10 to 1.04), but showed a significant improvement compared with Sham ESWT (MD −1.48; 95% CI −2.45 to −0.52). In addition, the Combined therapy was significantly superior to ESWT (MD -0.88; 95% CI -1.40 to -0.35) and CPT (MD −0.90; 95% CI −1.12 to −0.67) (**Figure 3**).

For ODI, ESWT was significantly more effective than CPT (MD −4.51; 95% CI −8.80 to −0.22), but it did not show a significant benefit over Sham ESWT (MD −0.49; 95% CI −1.20 to 0.22). In addition, Combined therapy was significantly superior to ESWT (MD −4.47; 95% CI −7.88 to −1.06) and CPT (MD −4.21; 95% CI −6.97 to −1.46) (**Figure 3**).

### 3.5 Comparing efficacy of outcomes

**Figure 4:**
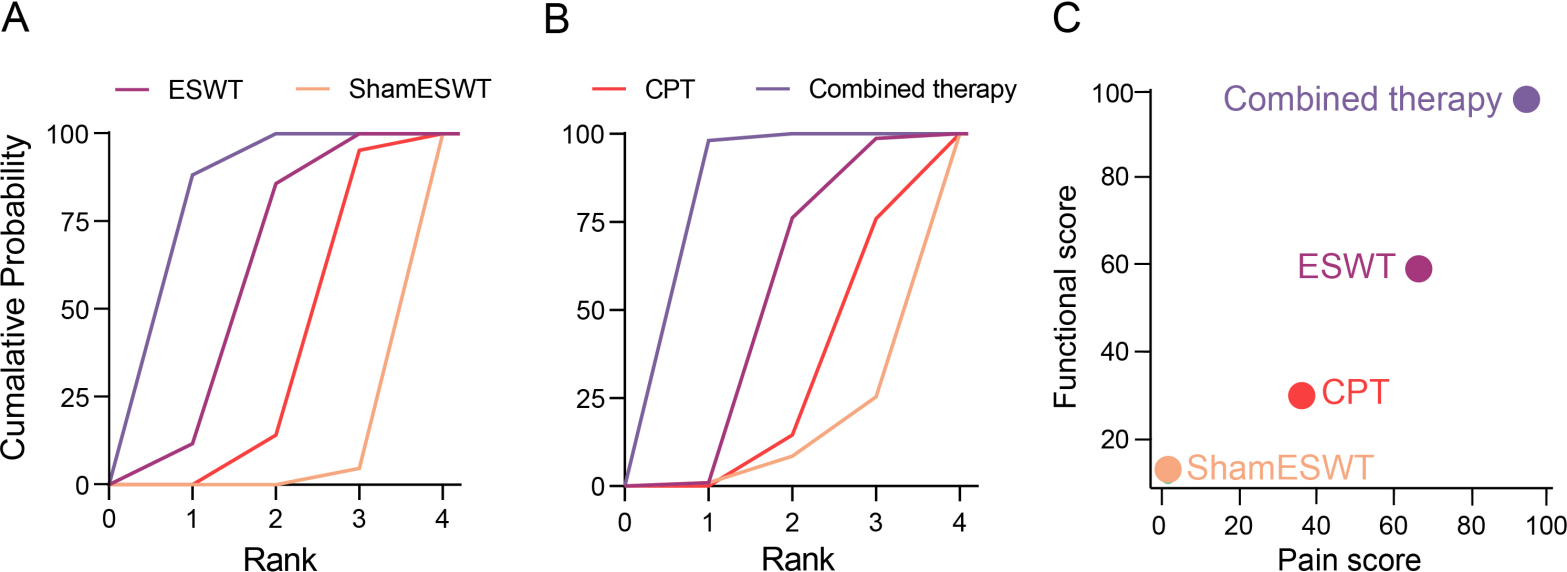
Overall SUCRA rankings for efficacy outcomes. The SUCRA plot of pain **(A)**, function **(B)**, and pairs efficacy outcomes of pain and function **(C)**. ESWT indicates extracorporeal shock wave therapy; Sham ESWT, sham extracorporeal shock wave therapy; CPT, Conventional physical therapy; Combined therapy, ESWT with conventional physical therapy.

The SUCRA plot of pain score showed that Combined therapy was ranked first (96.0%), followed by ESWT (66.3%), CPT (36.1%), and Sham ESWT (1.6%). The SUCRA plot of functional score showed that Combined therapy was ranked first (99.3%), followed by ESWT (59.0%), CPT (30.1%), and Sham ESWT (11.6%) (**Figure 4**).

### 3.6 Bias and quality of evidence ROB

**Figure 5:**
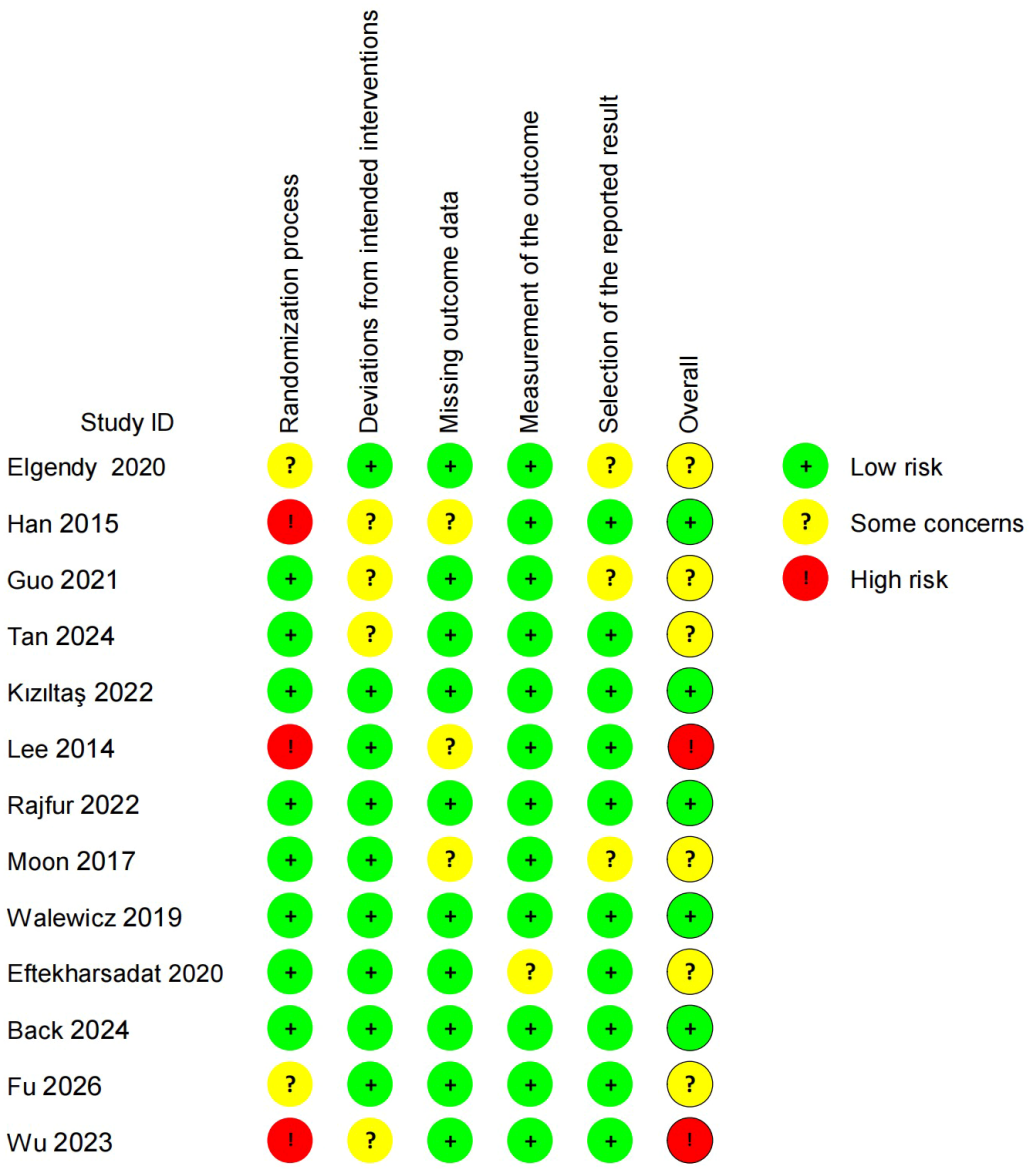
Study quality assessment by ROB 2.0.

Of the 14 included studies, 5 were judged to be at low overall risk of bias, 6 raised some concerns, and 3 were judged to be at high risk of bias. At the domain level, 9 studies (64.3%) were rated as low risk for bias arising from the randomization process; 10 (71.4%) for bias due to deviations from intended interventions; 13 (92.9%) for bias due to missing outcome data; 11 (78.6%) for bias in measurement of the outcome; and 5 (35.7%) for bias in selection of the reported result (**Figure 5**; **Supplementary Figures 5**, **6**).

### 3.7 Certainty of evidence

Certainty of evidence was assessed for 12 comparisons. Eight comparisons were informed by mixed evidence, and four were based on indirect evidence only. Among mixed-evidence comparisons, six were rated as low certainty and two as very low certainty; all indirect-only comparisons were rated as very low certainty.

### 3.8 Inconsistency test

No inconsistencies were identified in ODI. However, the direct pairwise estimate for ESWT versus CPT differed from the corresponding mixed network estimate, suggesting that the functional results may have been influenced by sparse network geometry and the contribution of indirect evidence rather than by statistical incoherence. No overall inconsistency was detected in pain scores, but local inconsistencies emerged for the Combined therapy and CPT, with the relevant comparisons downgraded in quality assessment. Sensitivity analyses for both direct pairwise and indirect comparisons (network meta-analysis) showed no evidence of heterogeneity. Results were consistent following exclusion of high-risk studies, supporting the robustness of our findings. While visualizations were provided for pairwise comparison analyses based on three or more studies (**Supplementary Figure 7**). The results from both analyses support the overall reliability of the treatment effects.

## 4 Discussion

This study conducted a network meta-analysis based on data from 14 randomized controlled trials involving a total of 738 CLBP patients. The analysis compared ESWT, Sham ESWT, CPT, and Combined therapy within a single evidence network. The results consistently showed that the Combined therapy held a significant advantage in both pain relief and functional improvement. ESWT demonstrated superior effectiveness in pain reduction compared to Sham ESWT. Furthermore, combined therapy outperformed CPT and sham ESWT in both pain relief and functional improvement, and was more effective than ESWT alone in enhancing functional outcomes. Notably, although combined therapy ranked highest in the SUCRA analysis, these ranking probabilities should not be interpreted as direct evidence of clinical superiority, because SUCRA reflects relative ranking rather than effect magnitude or clinical importance.

CLBP is thought to involve a multi-level pathological cycle maintained by peripheral tissue injury, spinal-central sensitization, movement control dysfunction, and fear-avoidance behaviors (Ferdinandov, 2024). This pathological complexity suggests that long-term improvement in CLBP requires multi-dimensional interventions rather than mere analgesia or isolated motor function enhancement. Yue et al. (2021) found that ESWT significantly reduced pain at the 1-month follow-up (SMD ≈ −0.81), confirming its robust short-term analgesic efficacy. Existing research indicates that ESWT primarily exerts biological effects through mechanical transduction, including transient pain relief, enhanced local microcirculation, promotion of angiogenesis, activation of nitric oxide and various growth factor pathways, and modulation of inflammatory mediators and pain-related neuropeptides (Simplicio et al., 2020; Huang et al., 2024). Our network comparative analysis showed no significant improvement in patients’ ODI scores, a finding further corroborated by a systematic review by Wu et al (Wu et al., 2023), which noted that ESWT’s effects on disability outcomes are either minimal or statistically insignificant.

CPT is widely recognized for its long-term benefits in improving muscle instability and motor dysfunction (Goubert et al., 2017; Anthierens et al., 2024). Specifically, CPT mitigates central sensitization and activates deep stabilizing muscles (e.g., multifidus, transversus abdominis), thereby promoting motor control retraining, enhancing quality of life, and reducing recurrence risk (Nayel et al., 2025; Tomschi et al., 2025). Nevertheless, Gilanyi et al. (2024) pointed out that CPT is constrained by poor patient adherence—especially in those with severe pain—while Hayden et al. (2021) noted that despite its long-term functional benefits, CPT’s short-term analgesic effect is suboptimal. Notably, pairwise meta-analysis suggested that ESWT was superior to CPT for ODI, whereas the network meta-analysis showed no significant difference. As no inconsistency was detected in the ODI network, this discrepancy may be related to the sparse network structure, the contribution of indirect evidence, and between-study heterogeneity in treatment protocols and follow-up duration. The CPT effect typically manifests at 8–12 weeks, indicating that such discrepancies arise from short-term observation (Hayden et al., 2021). Consistently, O’Keeffe et al. (2020) reported that compared with group-based exercise and educational interventions, CPT reduced disability at 6 and 12 months but failed to alleviate pain—aligning with the earlier observation that CPT’s short-term analgesic effect is suboptimal. It should be noted that there is some heterogeneity among the intervention components of CPT. In addition, cautious interpretation is warranted for ODI findings, particularly in the comparison between ESWT and CPT, given the MCID threshold of 10 points (Ostelo and de Vet, 2005).

Against this backdrop, Jenkins et al. (2025) proposed a complementary therapeutic model for combined treatments: ESWT targets peripheral tissue injury to achieve rapid analgesia, while CPT exerts central regulatory effects to address sensitization and motor dysfunction. Some studies have demonstrated that combining ESWT with a structured exercise or rehabilitation program in patients with CLBP can not only achieve rapid pain relief but also contribute to improvements in functional outcomes. For example, in a randomized controlled trial by Taheri et al., the addition of ESWT to an exercise program and oral medication resulted in significantly greater short-term reductions in pain intensity (VAS) and disability (ODI) compared with sham ESWT combined with the same exercise and medication regimen (Taheri et al., 2021). Furthermore, other research suggests that, relative to conventional exercise or CPT alone, ESWT supplemented with exercise may yield more pronounced improvements in pain and dynamic balance, implying that the short-term biological effects of ESWT may provide a more favorable foundation for subsequent exercise-based rehabilitation (Lee et al., 2014).

This mechanism-driven combination directly addresses the multi-level pathology of CLBP, and accumulating evidence supports its feasibility (Karagiannopoulou et al., 2025; Pehlivanoglu et al., 2025). ESWT can rapidly attenuate pain and reduce tissue stress through mechanical transduction and local biological effects, which may enhance patients’ tolerance to subsequent CPT interventions (Wu et al., 2023). In contrast, CPT and structured exercise therapy are aimed at remodeling central sensitization, improving movement control, and addressing muscle instability through long-term neuromuscular adaptation (Tuninetti et al., 2025). The complementary mechanisms of these two modalities allow for more comprehensive coverage of the multifactorial pathophysiology underlying CLBP, thereby increasing the likelihood of sustained improvements in functional capacity and quality of life compared with single-modality interventions. For instance, Walewicz et al. (2019) observed that radial ESWT combined with CPT significantly improved both pain and function in CLBP patients—consistent with our findings that ESWT alone exerts significant analgesic effects (Korakakis et al., 2018; Baroncini et al., 2025), and that the synergistic combination of ESWT and CPT effectively overcomes the inherent limitations of each monotherapy. Notably, this network meta-analysis integrated diverse ESWT protocols and standardized outcomes addressing the prior research gap of inconsistent combined therapy efficacy due to heterogeneous intervention.

## 5 Study limitations

Although this study has provided preliminary insights into the specific effects of ESWT through a detailed classification of intervention protocols, several limitations need to be addressed. First, despite integrating data from multiple studies through network meta-analysis, the relatively small sample sizes and the heterogeneity between studies pose challenges. Key factors like the type of ESWT, energy intensity, and frequency were not standardized, which may limit the broader applicability of the findings (Kisch et al., 2015; Kraemer et al., 2016). Moreover, due to the limited number of studies, the CPT node was not further subdivided, which led to local inconsistency. Future research should continue to explore the specific effects of different control groups. Second, patients with CLBP are a highly diverse group, yet most studies do not categorize treatment outcomes based on subtypes such as myofascial or discogenic pain. Future research should focus on dividing patients into these subtypes and investigating how different subtypes respond to combined treatments. This approach could help in developing more personalized treatment plans (Yahata et al., 2016). Lastly, since the included studies mainly had short follow-up periods, future research should aim for longer follow-up times to better evaluate the long-term effects of combined treatments on pain relief, functional recovery, and the prevention of recurrence.

## 6 Conclusion

Based on the low to very low-certainty evidence, combined therapy shows potential for improving both pain and function in patients with CLBP, whereas ESWT alone may reduce pain but its effect on functional outcomes remains uncertain. Further high-quality, large-scale RCTs are needed to confirm these findings.

## Data Availability

The original contributions presented in the study are included in the article/**Supplementary Material**. Further inquiries can be directed to the corresponding authors.

## References

[B1] Anthierens A. Thevenon A. Olivier N. Mucci P. (2024). Paraspinal muscle oxygenation and mechanical efficiency are reduced in individuals with chronic low back pain. Sci. Rep. 14, 4943. doi: 10.1038/s41598-024-55672-8 38418858 PMC10901808

[B2] Back C. G. N. Peron R. Lopes C. V. R. de Souza J. V. E. Liebano R. E. (2024). Immediate effect of extracorporeal shockwave therapy in patients with chronic non-specific low back pain: a randomised placebo-controlled triple-blind trial. Clin. Rehabil. 38, 1080–1090. doi: 10.1177/02692155241251844 38710199

[B3] Baran T. M. Lin F. V. Geha P. (2022). Functional brain mapping in patients with chronic back pain shows age-related differences. Pain 163, e917–e926. doi: 10.1097/j.pain.0000000000002534 34799532

[B4] Baroncini A. Maffulli N. Manocchio N. Bossa M. Foti C. Schäfer L. . (2025). Active and passive physical therapy in patients with chronic low-back pain: a level I Bayesian network meta-analysis. J. Orthopaedics Traumatol 26, 66. doi: 10.1186/s10195-025-00885-4 41042338 PMC12494532

[B5] Brozek J. L. Canelo-Aybar C. Akl E. A. Bowen J. M. Bucher J. Chiu W. A. . (2021). GRADE Guidelines 30: the GRADE approach to assessing the certainty of modeled evidence-an overview in the context of health decision-making. J. Clin. Epidemiol. 129, 138–150. doi: 10.1016/j.jclinepi.2020.09.018 32980429 PMC8514123

[B6] Burton I. (2022). Combined extracorporeal shockwave therapy and exercise for the treatment of tendinopathy: a narrative review. Sports Med. Health Sci. 4, 8–17. doi: 10.1016/j.smhs.2021.11.002 35782779 PMC9219268

[B7] Cashin A. G. Furlong B. M. Kamper S. J. De Carvalho D. MaChado L. A. Davidson S. R. . (2025). Analgesic effects of non-surgical and non-interventional treatments for low back pain: a systematic review and meta-analysis of placebo-controlled randomised trials. BMJ Evidence-Based Med. 1–15. doi: 10.1136/bmjebm-2024-112974 40101974

[B8] Eftekharsadat B. Fasaie N. Golalizadeh D. Babaei-Ghazani A. Jahanjou F. Eslampoor Y. . (2020). Comparison of efficacy of corticosteroid injection versus extracorporeal shock wave therapy on inferior trigger points in the quadratus lumborum muscle: a randomized clinical trial. BMC Musculoskeletal Disord. 21, 695. doi: 10.1186/s12891-020-03714-3 33076888 PMC7574569

[B9] Elgendy M. Mohamed M. Hussien H. (2020). Effect of extracorporeal shock wave on electromyographic activity of trunk muscles in non- specific chronic low back pain: a randomized controlled trial. Eurasian J. Biosci. 14, 6955–6962.

[B10] Ferdinandov D. (2024). Focused extracorporeal shockwave therapy for the treatment of low back pain: a systematic review. Front. Med. 11, 1435504. doi: 10.3389/fmed.2024.1435504 39267973 PMC11390445

[B11] Fu Y. S. Shih K. S. Lin Y. T. Hsieh L. F. Liu Y. F. Chen Y. R. (2026). Efficacy of ultrasound-guided piriformis muscle corticosteroid injection versus extracorporeal shockwave therapy in patients with piriformis syndrome: a randomized controlled trial. J. Formos. Med. Assoc. 125 (4), 391–397. doi: 10.1016/j.jfma.2025.01.020 40016058

[B12] GBD 2021 Low Back Pain Collaborators . (2023). Global, regional, and national burden of low back pain, 1990-2020, its attributable risk factors, and projections to 2050: a systematic analysis of the Global Burden of Disease Study 2021. Lancet Rheumatol. 5 (6), e316–e329. doi: 10.1016/s2665-9913(23)00098-x 37273833 PMC10234592

[B13] Gilanyi Y. L. Shah B. Cashin A. G. Gibbs M. T. Bellamy J. Day R. . (2024). Barriers and enablers to exercise adherence in people with nonspecific chronic low back pain: a systematic review of qualitative evidence. Pain 165, 2200–2214. doi: 10.1097/j.pain.0000000000003234 38635470 PMC11404330

[B14] Goubert D. De Pauw R. Meeus M. Willems T. Cagnie B. Schouppe S. . (2017). Lumbar muscle structure and function in chronic versus recurrent low back pain: a cross-sectional study. Spine J. 17, 1285–1296. doi: 10.1016/j.spinee.2017.04.025 28456669

[B15] Guo X. Li L. Yan Z. Li Y. Peng Z. Yang Y. . (2021). Efficacy and safety of treating chronic nonspecific low back pain with radial extracorporeal shock wave therapy (rESWT), rESWT combined with celecoxib and eperisone (C + E) or C + E alone: a prospective, randomized trial. J. Orthopaedic Surg. Res. 16, 705. doi: 10.1101/2020.11.28.20240119 34863239 PMC8642949

[B16] Guyatt G. Oxman A. D. Akl E. A. Kunz R. Vist G. Brozek J. . (2011). GRADE guidelines: 1. Introduction-GRADE evidence profiles and summary of findings tables. J. Clin. Epidemiol. 64, 383–394. doi: 10.1016/j.jclinepi.2010.04.026 21195583

[B17] Han H. Lee D. Lee S. Jeon C. Kim T. (2015). The effects of extracorporeal shock wave therapy on pain, disability, and depression of chronic low back pain patients. J. Phys. Ther. Sci. 27 (2), 397–399. doi: 10.1589/jpts.27.397 25729177 PMC4339147

[B18] Hayden J. A. Ellis J. Ogilvie R. Malmivaara A. van Tulder M. W. (2021). Exercise therapy for chronic low back pain. Cochrane Database System Rev. 9, Cd009790. doi: 10.1002/14651858.cd009790 34580864 PMC8477273

[B19] Huang M. Shao H. Zhang S. Gao H. Feng S. Sun L. . (2024). Single-dose radial extracorporeal shock wave therapy modulates inflammation during meniscal tear healing in the avascular zone. Am. J. Sports Med. 52, 710–720. doi: 10.1177/03635465231221725 38353544

[B20] Jenkins H. J. Corrêa L. Brown B. T. Ferreira G. E. Nim C. Aspinall S. L. . (2025). Long-term effectiveness of non-surgical interventions for chronic low back pain: a systematic review and meta-analysis. Lancet Rheumatol. 7, e607–e617. doi: 10.1016/s2665-9913(25)00064-5 40449512

[B21] Karagiannopoulou V. Meirezonne H. De Greef I. Van Oosterwijck J. Matheve T. Danneels L. . (2025). The effects of exercise therapy on lumbar muscle structure in low back pain: A systematic review and meta-analysis. Ann. Phys. Rehabil. Med. 68, 101988. doi: 10.1016/j.rehab.2025.101988 40311262

[B22] Kisch T. Sorg H. Forstmeier V. Knobloch K. Liodaki E. Stang F. . (2015). Remote effects of extracorporeal shock wave therapy on cutaneous microcirculation. J. Tissue Viabil 24, 140–145. doi: 10.1016/j.jtv.2015.07.004 26299636

[B23] Kızıltaş Ö. Okçu M. Tuncay F. Koçak F. A. (2022). Comparison of the effectiveness of conventional physical therapy and extracorporeal shock wave therapy on pain, disability, functional status, and depression in patients with chronic low back pain. Turk J. Phys. Med. Rehabil 68, 399–408. doi: 10.5606/tftrd.2022.8905 36475112 PMC9706798

[B24] Kong L. Tian X. Yao X. (2023). Effects of extracorporeal shock wave therapy on chronic low back pain and quality of life. Minerva Surg. 78, 305–306. doi: 10.23736/s2724-5691.22.09537-5 35420285

[B25] Korakakis V. Whiteley R. Tzavara A. Malliaropoulos N. (2018). The effectiveness of extracorporeal shockwave therapy in common lower limb conditions: a systematic review including quantification of patient-rated pain reduction. Br. J. Sports Med. 52, 387–407. doi: 10.1136/bjsports-2016-097347 28954794

[B26] Kraemer R. Sorg H. Forstmeier V. Knobloch K. Liodaki E. Stang F. H. . (2016). Immediate dose-response effect of high-energy versus low-energy extracorporeal shock wave therapy on cutaneous microcirculation. Ultrasound Med. Biol. 42, 2975–2982. doi: 10.1016/j.ultrasmedbio.2016.08.010 27662701

[B27] Lee S. Lee D. Park J. (2014). Effects of extracorporeal shockwave therapy on patients with chronic low back pain and their dynamic balance ability. J. Phys. Ther. Sci. 26 (1), 7–10. doi: 10.1589/jpts.26.7 24567665 PMC3927045

[B28] Liu K. Zhang Q. Chen L. Zhang H. Xu X. Yuan Z. . (2023). Efficacy and safety of extracorporeal shockwave therapy in chronic low back pain: a systematic review and meta-analysis of 632 patients. J. Orthopaedic Surg. Res. 18, 455. doi: 10.1186/s13018-023-03943-x 37355623 PMC10290808

[B29] Moon Y. E. Seok H. Kim S. H. Lee S. Y. Yeo J. H. (2017). Extracorporeal shock wave therapy for sacroiliac joint pain: a prospective, randomized, sham-controlled short-term trial. J. Back Musculoskelet. Rehabil 30, 779–784. doi: 10.3233/bmr-150405 28372309

[B30] Nayel N. Ezzat H. Ahmed S. Saleh H. (2025). Effect of core stability exercises and Russian electrical stimulation on nonspecific low back pain: a single-blinded randomized controlled trial. Sci. Rep. 15, 44053. doi: 10.1038/s41598-025-28313-x 41407791 PMC12715220

[B31] Nedelka T. Katolicky J. Nedelka J. Hobrough P. Knobloch K. (2025). Efficacy of high-energy, focused ESWT in treatment of lumbar facet joint pain: a randomized sham-controlled trial. Int. J. Surg. (Lond Engl) 111, 4177–4186. doi: 10.1097/js9.0000000000002538 40391994

[B32] Nevelikova M. Zlamal F. Dosbaba F. Su J. J. Batalik L. (2025). Motivation to exercise in patients with chronic low back pain. BMC Musculoskeletal Disord. 26, 226. doi: 10.1186/s12891-025-08461-x 40050856 PMC11883927

[B33] Nicol V. Verdaguer C. Daste C. Bisseriex H. Lapeyre É. Lefèvre-Colau M. M. . (2023). Chronic low back pain: a narrative review of recent international guidelines for diagnosis and conservative treatment. J. Clin. Med. 12 (4), 1685. doi: 10.3390/jcm12041685 36836220 PMC9964474

[B34] O’Keeffe M. O’Sullivan P. Purtill H. Bargary N. O’Sullivan K. (2020). Cognitive functional therapy compared with a group-based exercise and education intervention for chronic low back pain: a multicentre randomised controlled trial (RCT). Br. J. Sports Med. 54, 782–789. doi: 10.1136/bjsports-2019-100780 31630089 PMC7361017

[B35] Ostelo R. W. J. G. de Vet H. C. W. (2005). Clinically important outcomes in low back pain. Best Pract. Res. Clin. Rheumatol. 19, 593–607. doi: 10.1016/j.berh.2005.03.003 15949778

[B36] Page M. J. McKenzie J. E. Bossuyt P. M. Boutron I. Hoffmann T. C. Mulrow C. D. . (2021). The PRISMA 2020 statement: an updated guideline for reporting systematic reviews. BMJ (Clin Res. Ed) 372, n71. doi: 10.31222/osf.io/v7gm2 33782057 PMC8005924

[B37] Pehlivanoglu G. Aydin C. G. Yildiz K. I. Albayrak K. Kurk M. B. Ozkul B. (2025). Comparison of isolated eccentric exercise and eccentric exercise combined with shock wave therapy in non-insertional Achilles tendinopathy. J. Foot Ankle Surg. 26:S1067-2516(25)00175-9. doi: 10.1053/j.jfas.2025.05.009 40578503

[B38] Qiao Z. Kou Z. Zhang J. Lv D. Cui X. Li D. . (2025). Optimal intensity and type of lower limb aerobic training for patients with chronic obstructive pulmonary disease: a systematic review and network meta-analysis of RCTs. Ther. Adv. Respir. Dis. 19, 17534666251323190. doi: 10.1177/17534666251323190 40083154 PMC11907633

[B39] Rajfur K. Rajfur J. Matusz T. Walewicz K. Dymarek R. Ptaszkowski K. . (2022). Efficacy of focused extracorporeal shock wave therapy in chronic low back pain: a prospective randomized 3-month follow-up study. Med. Sci. Monit: Int. Med. J. Exp. Clin. Res. 28, e936614. doi: 10.12659/msm.936614 35689370 PMC9199449

[B40] Rizzo R. R. Cashin A. G. Wand B. M. Ferraro M. C. Sharma S. Lee H. . (2025). Non-pharmacological and non-surgical treatments for low back pain in adults: an overview of Cochrane reviews. Cochrane Database System Rev. 3, Cd014691. doi: 10.1002/14651858.cd014691.pub2 40139265 PMC11945228

[B41] Schneider R. (2018). Effectiveness of myofascial trigger point therapy in chronic back pain patients is considerably increased when combined with a new, integrated, low-frequency shock wave vibrotherapy (Cellconnect Impulse): a two-armed, measurement repeated, randomized, controlled pragmatic trial. J. Back Musculoskeletal Rehabil. 31, 57–64. doi: 10.3233/bmr-169662 28826166

[B42] Simplicio C. L. Purita J. Murrell W. Santos G. S. Dos Santos R. G. Lana J. (2020). Extracorporeal shock wave therapy mechanisms in musculoskeletal regenerative medicine. J. Clin. Orthopaedics Trauma 11, S309–S318. doi: 10.1016/j.jcot.2020.02.004 32523286 PMC7275282

[B43] Sterne J. A. C. Savović J. Page M. J. Elbers R. G. Blencowe N. S. Boutron I. . (2019). RoB 2: a revised tool for assessing risk of bias in randomised trials. BMJ (Clin Res. Ed) 366, l4898. doi: 10.1136/bmj.l4898 31462531

[B44] Taheri P. Khosrawi S. Ramezani M. (2021). Extracorporeal shock wave therapy combined with oral medication and exercise for chronic low back pain: a randomized controlled trial. Arch. Phys. Med. Rehabil. 102, 1294–1299. doi: 10.1016/j.apmr.2020.12.008 33453192

[B45] Tan K. L. Wang R. Liu J. J. Peng Y. Li H. Li C. Y. (2024). Effectiveness of focused extracorporeal shock wave versus manual therapy in postpartum patients with sacroiliac joint dysfunction: a prospective clinical trial. J. Orthopaedic Surg. Res. 19 (1), 28. doi: 10.1186/s13018-023-04491-0 38172900 PMC10763479

[B46] Tenforde A. S. Borgstrom H. E. DeLuca S. McCormack M. Singh M. Hoo J. S. . (2022). Best practices for extracorporeal shockwave therapy in musculoskeletal medicine: clinical application and training consideration. PM R. J. Injury Funct Rehabil. 14, 611–619. doi: 10.1002/pmrj.12790 35187851 PMC9321712

[B47] Tieppo A. M. Tieppo J. S. Rivetti L. A. (2024). Analysis of intestinal bacterial microbiota in individuals with and without chronic low back pain. Current Issues in Molecular Biology 46 (7), 7339–7352. doi: 10.3390/cimb4607043 39057076 PMC11276315

[B48] Tomschi F. Zschunke A. Hilberg T. (2025). Ten minutes of core stabilisation exercise result in local exercise-induced hypoalgesia in patients with chronic unspecific low back pain. Eur. J. Pain (Lond Engl) 29, e4794. doi: 10.1002/ejp.4794 39923121 PMC11807238

[B49] Tuninetti A. Barbari V. Storari L. Bisconti M. Piano L. Dunning J. . (2025). Therapeutic exercise progression in patients with nonspecific low back pain: A systematic review. J. Pain Res. 18, 6397–6407. doi: 10.2147/jpr.s539160 41356704 PMC12676137

[B50] Walewicz K. Taradaj J. Rajfur K. Ptaszkowski K. Kuszewski M. T. Sopel M. . (2019). The effectiveness of radial extracorporeal shock wave therapy in patients with chronic low back pain: a prospective, randomized, single-blinded pilot study. Clin. Interventions Aging 14, 1859–1869. doi: 10.2147/cia.s224001 31806944 PMC6857735

[B51] Wang S. Huang S. Xu X. Liu R. (2024). Effects of radial extracorporeal shock wave with different frequencies on acute skeletal muscle injury in rabbits. Sci. Rep. 14, 21276. doi: 10.1038/s41598-024-72371-6 39261623 PMC11391075

[B52] Wu T. Wang D. Zhang X. Li J. Yuan B. (2023). Comparison of pain relief and limb function improvement after extracorporeal shock wave therapy and thermomagnetic therapy in the treatment of low back pain. Pakistan J. Med. Sci. 39 (1), 268–273. doi: 10.12669/pjms.39.1.6668 36694762 PMC9842974

[B53] Wu Z. Zhou T. Ai S. (2023). Extracorporeal shock wave therapy for low back pain: a systematic review and meta-analysis. Medicine 102, e36596. doi: 10.1097/md.0000000000036596 38206739 PMC10754595

[B54] Yahata K. Kanno H. Ozawa H. Yamaya S. Tateda S. Ito K. . (2016). Low-energy extracorporeal shock wave therapy for promotion of vascular endothelial growth factor expression and angiogenesis and improvement of locomotor and sensory functions after spinal cord injury. J. Neurosurg. Spine 25, 745–755. doi: 10.3171/2016.4.spine15923 27367940

[B55] Yue L. Sun M. S. Chen H. Mu G. Z. Sun H. L. (2021). Extracorporeal shockwave therapy for treating chronic low back pain: a systematic review and meta-analysis of randomized controlled trials. BioMed. Res. Int. 2021, 5937250. doi: 10.1155/2021/5937250 34840977 PMC8617566

[B56] Zhai T. Jiang F. Chen Y. Wang J. Feng W. (2024). Advancing musculoskeletal diagnosis and therapy: a comprehensive review of trigger point theory and muscle pain patterns. Front. Med. 11, 1433070. doi: 10.3389/fmed.2024.1433070 39050541 PMC11266154

[B57] Zhang B. Liu M. Bai Z. Shi L. Zhang J. Gao Y. (2025). Analysis of combined shockwave therapy and aquatic exercise for chronic nonspecific low back pain. Medicine 104, e43176. doi: 10.1097/md.0000000000043176 40660599 PMC12262937

[B58] Zhang X. Ma Y. (2023). Global trends in research on extracorporeal shock wave therapy (ESWT) from 2000 to 2021. BMC Musculoskeletal Disord. 24, 312. doi: 10.1186/s12891-023-06407-9 37081473 PMC10116688

[B59] Zheng P. Scheffler A. Ewing S. Hue T. F. Jones Berkeley S. Morshed S. . (2025). Chronic low back pain causal risk factors identified by Mendelian randomization: a cross-sectional cohort analysis. Spine J: Off. J. North Am. Spine Soc. 25, 1154–1166. doi: 10.1016/j.spinee.2024.12.029 39818276 PMC13477212

