# Supplementary material for: Efficacy of extracorporeal shock wave therapy combined with conventional physical therapy for chronic low back pain: a systematic review and network meta-analysis of randomized controlled trials
Source: Front Physiol. 2026 Jun 10;17:1807929. doi: 10.3389/fphys.2026.1807929 (PMC13290623; doi:10.3389/fphys.2026.1807929)
Supplement: Supplementary file 1 [file DataSheet1.docx]

**Supplemental materials content**

**Part A**

Search strategy

**Part B**

**Table S1** Details and parameters of ESWT

**Figure S1** Network meta-analysis contribution plot of pain score (VAS and NRS)

**Figure S2** Network meta-analysis contribution plot of ODI

**Figure S3** Comparison of treatment effects on the pain score (VAS and NRS)

**Figure S4** Comparison of treatment effects on the ODI

**Figure S5** Network meta-analysis funnel plots for the pain score (VAS and NRS)

**Figure S6** Network meta-analysis funnel plots for ODI

**Part A**

**Search strategy**

**The search results of each database are as follows: 17 results in PubMed; 26 results in Web of Science; 124 results in Cochrane；38 results in Embase; 24 results in Clinicaltrials.gov**

**Pubmed**

#1 "Low Back Pain"[Mesh] OR Low Back Pain[Title/Abstract] OR Mechanical Low Back Pain[Title/Abstract] OR Low Back Pain, Mechanical[Title/Abstract] OR Low Back Pain, Postural[Title/Abstract] OR Postural Low Back Pain[Title/Abstract] OR Low Back Pain, Posterior Compartment[Title/Abstract] OR Recurrent Low Back Pain[Title/Abstract] OR Low Back Pain, Recurrent[Title/Abstract] OR Lower Back Pains[Title/Abstract] OR Lower Back Pain[Title/Abstract] OR Back Pain, Low[Title/Abstract] OR Back Ache, Low[Title/Abstract] OR Pains, Lower Back[Title/Abstract]

#2 "Extracorporeal Shockwave Therapy"[Mesh] OR Extracorporeal Shockwave Therapy[Title/Abstract] OR Therapy, Shock Wave[Title/Abstract] OR Extracorporeal Shock Wave Therapy[Title/Abstract] OR Shock Wave Therapy[Title/Abstract] OR Shock Wave Therapies[Title/Abstract] OR Shockwave Therapies, Extracorporeal[Title/Abstract] OR Therapy, Extracorporeal Shockwave[Title/Abstract] OR Extracorporeal Shockwave Therapies[Title/Abstract] OR Shockwave Therapy, Extracorporeal[Title/Abstract]

#3: #1 AND #2 ((Low Back Pain[Title/Abstract] OR Mechanical Low Back Pain[Title/Abstract] OR Low Back Pain, Mechanical[Title/Abstract] OR Low Back Pain, Postural[Title/Abstract] OR Postural Low Back Pain[Title/Abstract] OR Low Back Pain, Posterior Compartment[Title/Abstract] OR Recurrent Low Back Pain[Title/Abstract] OR Low Back Pain, Recurrent[Title/Abstract] OR Lower Back Pains[Title/Abstract] OR Lower Back Pain[Title/Abstract] OR Back Pain, Low[Title/Abstract] OR Back Ache, Low[Title/Abstract] OR Pains, Lower Back[Title/Abstract])) AND ((Extracorporeal Shockwave Therapy[MeSH Terms]) OR (Extracorporeal Shockwave Therapy[Title/Abstract] OR Therapy, Shock Wave[Title/Abstract] OR Extracorporeal Shock Wave Therapy[Title/Abstract] OR Shock Wave Therapy[Title/Abstract] OR Shock Wave Therapies[Title/Abstract] OR Shockwave Therapies, Extracorporeal[Title/Abstract] OR Therapy, Extracorporeal Shockwave[Title/Abstract]))

We have found **42** articles that used the keywords above.

**Web of Sciense**

#1: TS=(Low Back Pain) OR AB=(Mechanical Low Back Pain) OR AB=(Low Back Pain, Mechanical) OR AB=(Low Back Pain, Postural) OR AB=(Postural Low Back Pain) OR AB=(Low Back Pain, Posterior Compartment) OR AB=(Recurrent Low Back Pain) OR AB=(Low Back Pain, Recurrent) OR AB=(Lower Back Pains) OR AB=(Lower Back Pain) OR AB=(Back Pain, Low) OR AB=(Back Ache, Low) OR AB=(Pains, Lower Back)

#2: TS=(Extracorporeal Shock Wave Therapy) OR AB=(Therapy, Shock Wave) OR AB=(Shock Wave Therapy) OR AB=(Shock Wave Therapies) OR AB=(Shockwave Therapies, Extracorporeal) OR AB=(Therapy, Extracorporeal Shockwave) OR AB=(Extracorporeal Shockwave Therapies) OR AB=(Shockwave Therapy, Extracorporeal)

#3: #1 AND #2 (TS=(Low Back Pain) OR AB=(Mechanical Low Back Pain) OR AB=(Low Back Pain, Mechanical) OR AB=(Low Back Pain, Postural) OR AB=(Postural Low Back Pain) OR AB=(Low Back Pain, Posterior Compartment) OR AB=(Recurrent Low Back Pain) OR AB=(Low Back Pain, Recurrent) OR AB=(Lower Back Pains) OR AB=(Lower Back Pain) OR AB=(Back Pain, Low) OR AB=(Back Ache, Low) OR AB=(Pains, Lower Back)) AND (AB=(Extracorporeal Shock Wave Therapy) OR AB=(Therapy, Shock Wave) OR AB=(Shock Wave Therapy) OR AB=(Shock Wave Therapies) OR AB=(Shockwave Therapies, Extracorporeal) OR AB=(Therapy, Extracorporeal Shockwave) OR AB=(Extracorporeal Shockwave Therapies) OR AB=(Shockwave Therapy, Extracorporeal))

We have found 54 articles that used the keywords above.

**Cochrane**

#1: ('Low Back Pain':ab,ti OR 'Mechanical Low Back Pain':ab,ti OR 'Low Back Pain, Mechanical':ab,ti OR 'Low Back Pain, Postural':ab,ti OR 'Postural Low Back Pain':ab,ti OR 'Low Back Pain, Posterior Compartment':ab,ti OR 'Low Back Pain, Recurrent':ab,ti OR 'Lower Back Pains':ab,ti OR 'Lower Back Pain':ab,ti OR 'Back Pain, Low':ab,ti OR 'Back Ache, Low':ab,ti OR 'Pains, Lower Back':ab,ti)

#2: ('Extracorporeal Shockwave Therapy':ab,ti OR 'Therapy, Shock Wave':ab,ti OR 'Shock Wave Therapy':ab,ti OR 'Shock Wave Therapies':ab,ti OR 'Shockwave Therapies, Extracorporeal':ab,ti OR 'Therapy, Extracorporeal Shockwave':ab,ti OR 'Extracorporeal Shockwave Therapies':ab,ti OR 'Shockwave Therapy, Extracorporeal':ab,ti)

#3: #1 AND #2 ('Low Back Pain':ab,ti OR 'Mechanical Low Back Pain':ab,ti OR 'Low Back Pain, Mechanical':ab,ti OR 'Low Back Pain, Postural':ab,ti OR 'Postural Low Back Pain':ab,ti OR 'Low Back Pain, Posterior Compartment':ab,ti OR 'Low Back Pain, Recurrent':ab,ti OR 'Lower Back Pains':ab,ti OR 'Lower Back Pain':ab,ti OR 'Back Pain, Low':ab,ti OR 'Back Ache, Low':ab,ti OR 'Pains, Lower Back':ab,ti) AND ('Extracorporeal Shockwave Therapy':ab,ti OR 'Therapy, Shock Wave':ab,ti OR 'Shock Wave Therapy':ab,ti OR 'Shock Wave Therapies':ab,ti OR 'Shockwave Therapies, Extracorporeal':ab,ti OR 'Therapy, Extracorporeal Shockwave':ab,ti OR 'Extracorporeal Shockwave Therapies':ab,ti OR 'Shockwave Therapy, Extracorporeal':ab,ti)

We have found 124 articles that used the keywords above.

**Embase**

#1: 'Low Back Pain':ab,ti OR 'Mechanical Low Back Pain':ab,ti OR 'Low Back Pain, Mechanical':ab,ti OR 'Low Back Pain, Postural':ab,ti OR 'Postural Low Back Pain':ab,ti OR 'Low Back Pain, Posterior Compartment':ab,ti OR 'Recurrent Low Back Pain':ab,ti OR ' Lower Back Pains ':ab,ti OR ' Lower Back Pain ':ab,ti OR ' Back Pain, Low ':ab,ti OR ' Back Ache, Low ':ab,ti OR ' Pains, Lower Back ':ab,ti

#2: 'Extracorporeal Shock Wave Therapy ':ab,ti OR ' Therapy, Shock Wave ':ab,ti OR ' Shock Wave Therapy ':ab,ti OR ' Shock Wave Therapies ':ab,ti OR ' Shockwave Therapies, Extracorporeal ':ab,ti OR ' Therapy, Extracorporeal Shockwave ':ab,ti OR ' Extracorporeal Shockwave Therapies ':ab,ti OR ' Shockwave Therapy, Extracorporeal':ab,ti

#3: ('Extracorporeal Shock Wave Therapy ':ab,ti OR ' Therapy, Shock Wave ':ab,ti OR ' Shock Wave Therapy ':ab,ti OR ' Shock Wave Therapies ':ab,ti OR ' Shockwave Therapies, Extracorporeal ':ab,ti OR ' Therapy, Extracorporeal Shockwave ':ab,ti OR ' Extracorporeal Shockwave Therapies ':ab,ti OR ' Shockwave Therapy, Extracorporeal':ab,ti) AND ('Extracorporeal Shock Wave Therapy ':ab,ti OR ' Therapy, Shock Wave ':ab,ti OR ' Shock Wave Therapy ':ab,ti OR ' Shock Wave Therapies ':ab,ti OR ' Shockwave Therapies, Extracorporeal ':ab,ti)

#3: #1 AND #2

We have found 38 articles that used the keywords above.

**Clinicaltrials.gov**

#1: ("low back pain" OR "chronic low back pain" OR "chronic nonspecific low back pain" OR lumbago)

#2: ("extracorporeal shock wave" OR "extracorporeal shockwave" OR "shock wave therapy" OR ESWT)

#3: #1 AND #2

We have found 24 articles that used the keywords above.

| **Part B**  **Table S1** Details and parameters of ESWT | | | | | |
| --- | --- | --- | --- | --- | --- |
| Author, year | Device | Type | EFD or Pressure | Frequency (Hz) | Pulses per session |
| Elgendy et al^[1]^, 2020 | HC Shock Wave | – | 0.10 mJ/mm² | 5 | 2000 |
| Han et al^[2]^, 2015 | VITERA | – | 0.01–0.16 mJ/mm² | 2.5 | 1000 |
| Guo et al^[3]^, 2021 | DolorClast | radial | Increase to patient tolerance | 15 | 4000 |
| Tan et al^[4]^, 2024 | FS10Pro | focused | 0.0298 mJ/mm² | – | 4000 |
| Kızıltaş et al^[5]^, 2022 | Modus Touch | radial | 2.8 bar | 10 | 2600 |
| Lee et al^[6]^, 2014 | JEST-2000 | – | 0.10 mJ/mm² | 5 | 2000 |
| Rajfur et al^[7]^, 2022 | Duolith SD1 | focused | 0.15 mJ/mm² | 4 | 1000 |
| Moon et al^[8]^, 2017 | Aries | focused | 0.09–0.25 mJ/mm² | 3 | 2000 |
| Walewicz et al^[9]^, 2019 | Pro Shock Waves | radial | 0.1 mJ/mm² | 5 | 2000 |
| Eftekharsadat et al^[10]^, 2020 | enPulsPro | radial | 0.1 mJ/mm²/min | 10–16 | 1500 |
| Back et al^[11]^, 2024 | THORK | radial | 100 mJ (total) | 5 | 2000 |
| Fu et al^[12]^, 2026 | F10G4 | focused | 0.456–0.882 mJ/mm² | – | 3500 |
| Wu et al^[13]^, 2023 | BHSW ballistic | radial/ballistic | 1.5–3.0 bar | 8–10 Hz | 2000 |
| Nedelka et al^[14]^, 2025 | Duolith SD1 | focused | 0.35 mJ/mm² | 4 | 1200 |

Note: ESWT, extracorporeal shockwave therapy; EFD, Energy Flux Density.


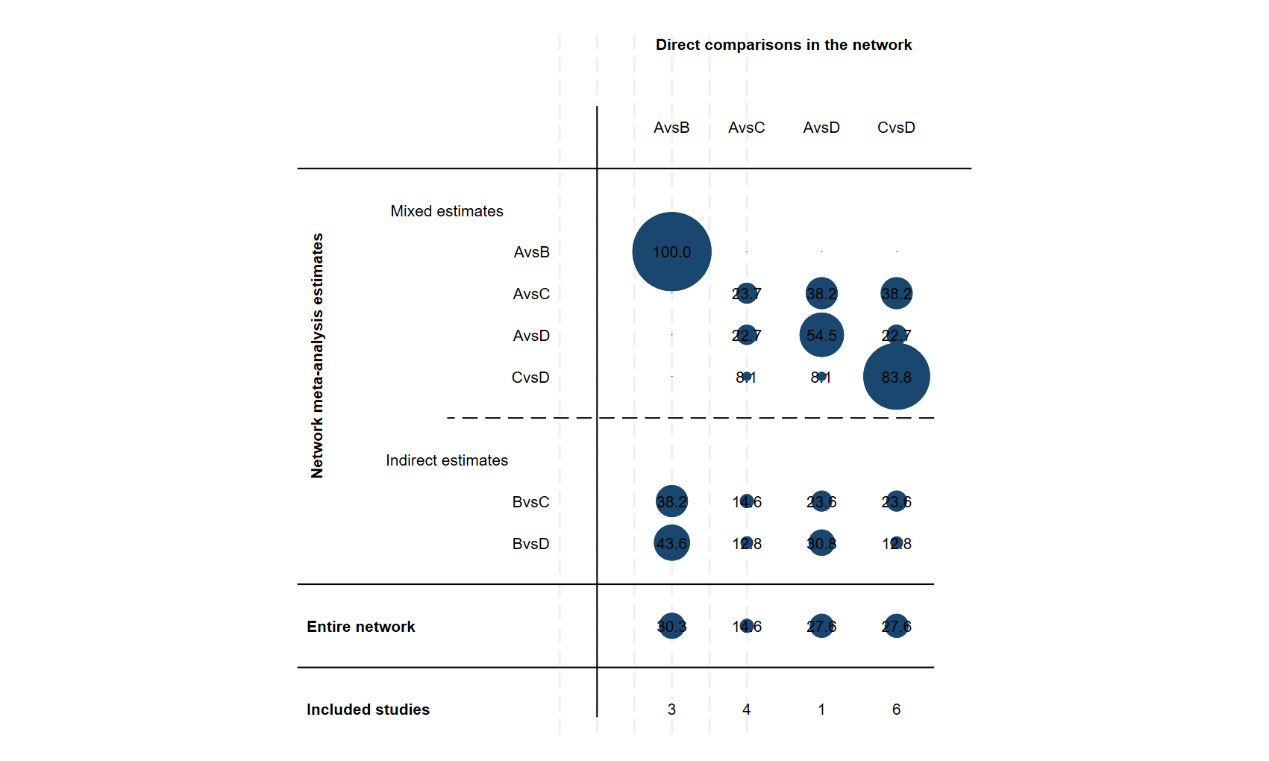


**Figure S1** Network meta-analysis contribution plot of pain score (VAS and NRS).


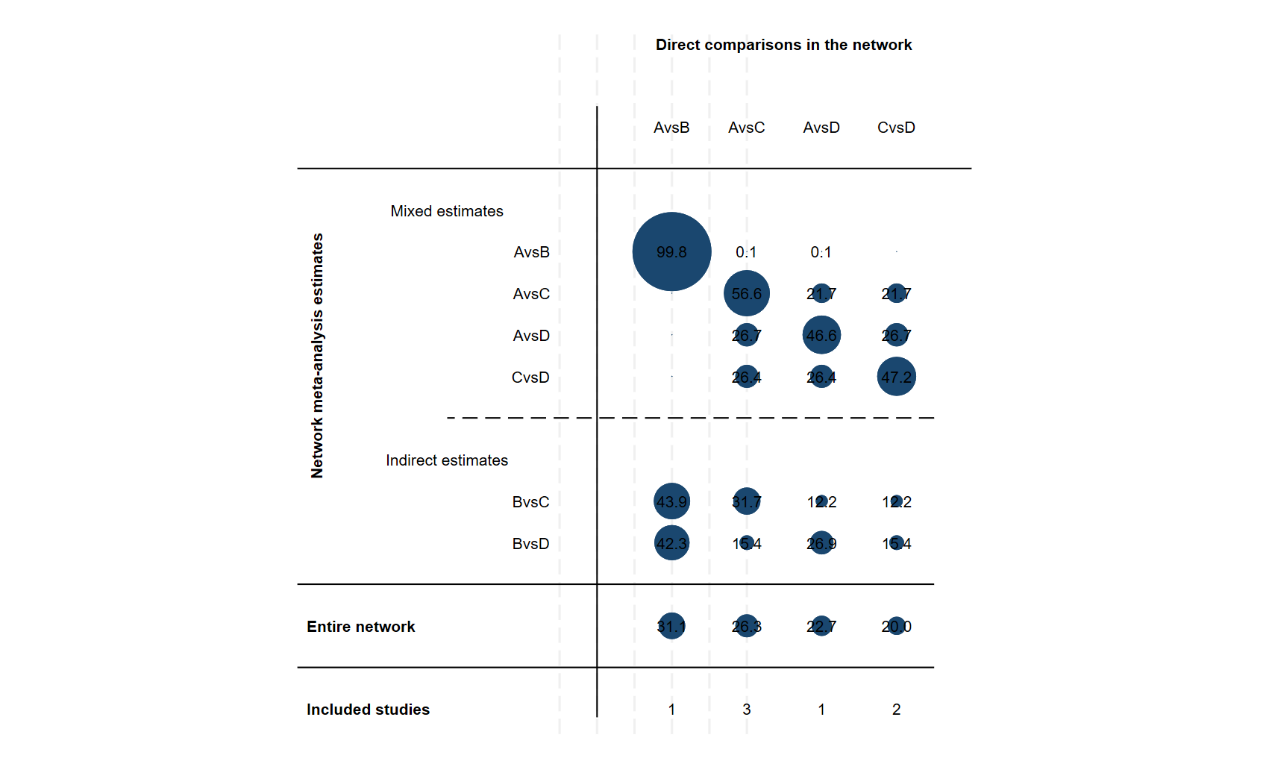


**Figure S2** Network meta-analysis contribution plot of ODI

| ESWT |  |  | |  | |  |
| --- | --- | --- | --- | --- | --- | --- |
| -1.22 (-1.86,-0.57) ^a,b^ | ShamESWT | |  | |  | |
| -0.27 (-0.74,-0.14) ^a,b,c^ | 0.53 (-0.33,1.38) ^a,b,c^ | | CPT | |  | |
| 0.26 (0.41,0.94) ^a,b^ | 1.48 (0.55,2.42) ^a,b,c^ | | 0.96 (0.49,1.42) ^a,b^ | | Combine | |

**Figure S3** Comparison of treatment effects on the pain score (VAS and NRS). Each value represents the result of comparing the intervention of that column vs the intervention of that row. 95% CIs that do not include 0 denote statistical significance. Yellow, and red signify low, and very low evidence.

^a^risk of bias

^b^Indirectness

^c^Imprecision

| ESWT |  |  |  |
| --- | --- | --- | --- |
| -4.00 (-9.7,1.70) ^a,b,c^ | ShamESWT |  |  |
| -2.08 (-4.95,0.78) ^a,b^ | 1.92 (-4.46,8.29) ^a,b,c^ | CPT |  |
| 3.84 (0.80,6.89) ^a,b,c^ | 7.84 (1.38,14.31) ^a,b,c^ | 5.93 (2.86, 9.00) ^a,b^ | Combine |

**Figure S4** Comparison of treatment effects on the ODI. Each value represents the result of comparing the intervention of that column vs the intervention of that row. 95% CIs that do not include 0 denote statistical significance. Yellow, and red signify low, and very low evidence.

^a^risk of bias

^b^Imprecision

^c^Indirectness.


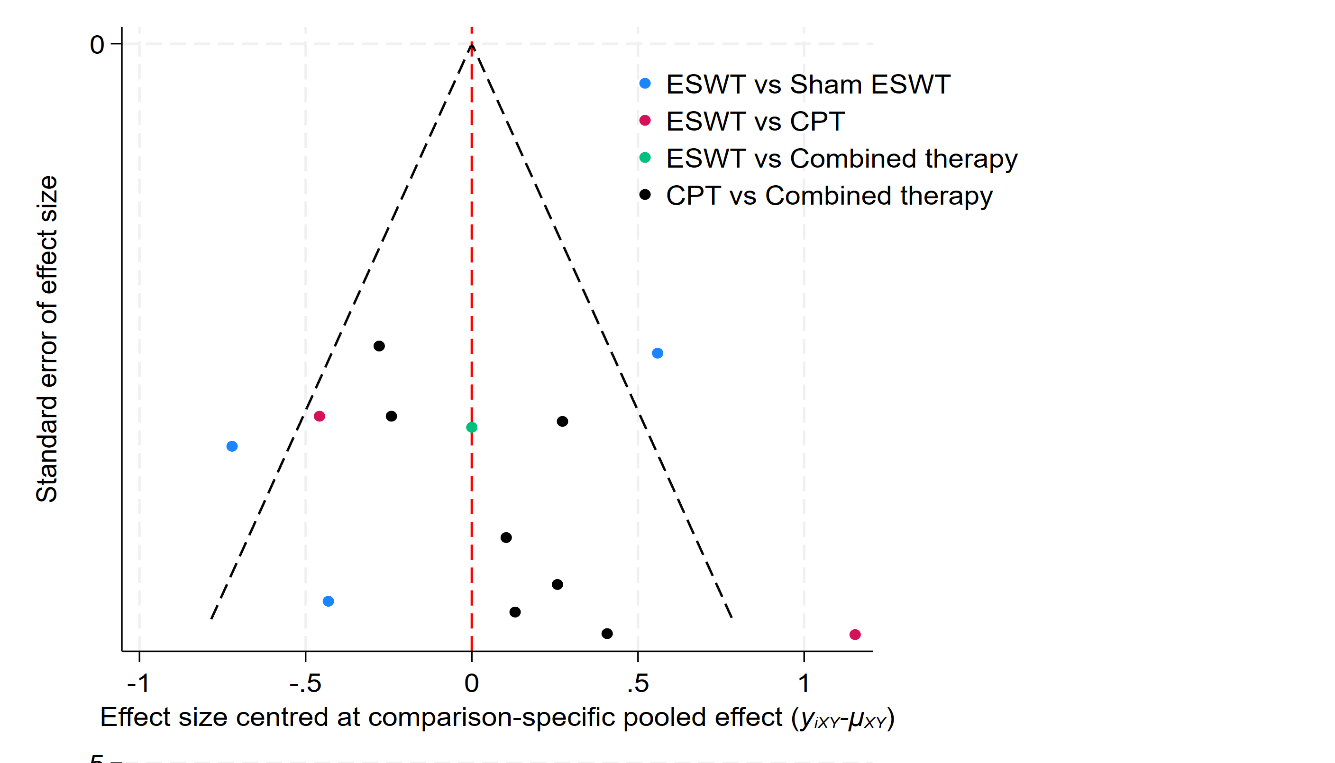


**Figure S5** Network meta-analysis funnel plots for the pain score (VAS and NRS). ESWT indicates extracorporeal shock wave therapy; ShamESWT, sham extracorporeal shock wave therapy; CPT, Conventional physical therapy; Combined therapy, ESWT with conventional physical therapy.


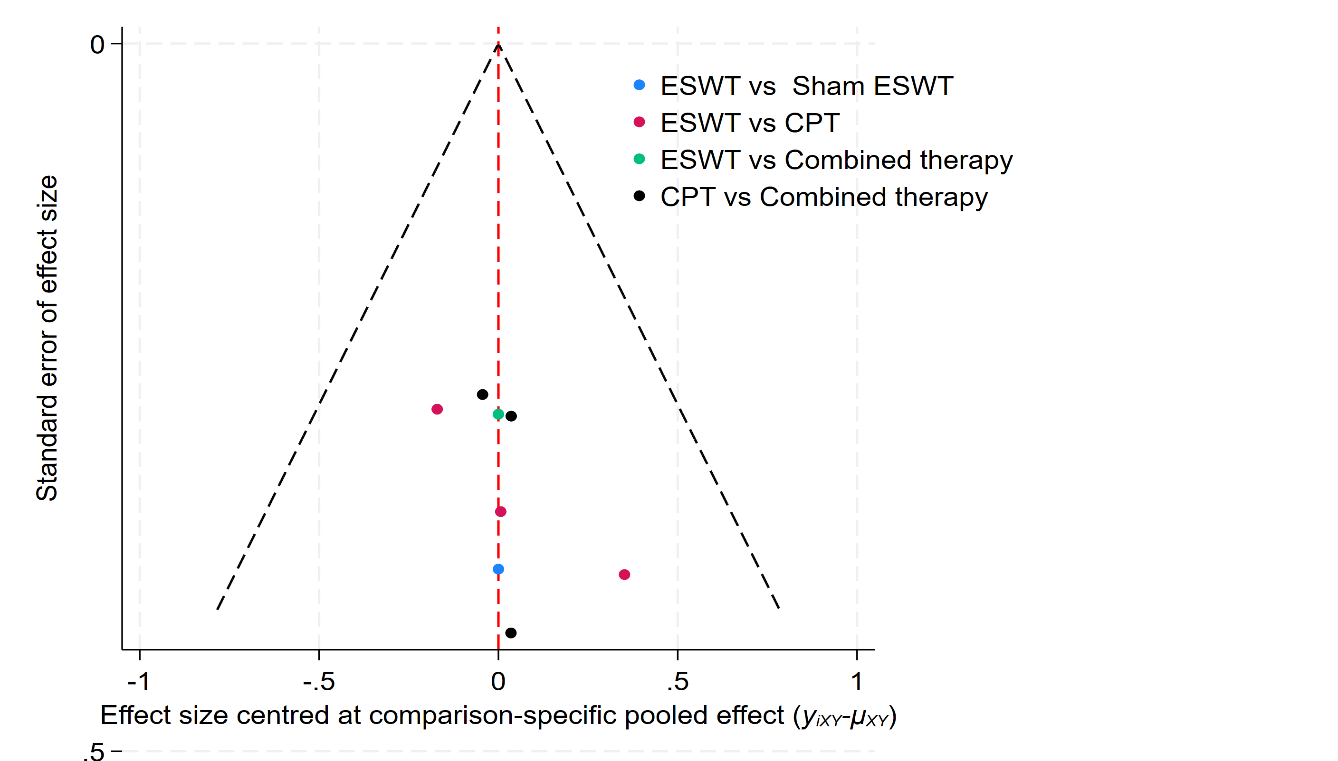


**Figure S6** Network meta-analysis funnel plots for the ODI. ESWT indicates extracorporeal shock wave therapy; ShamESWT, sham extracorporeal shock wave therapy; CPT, Conventional physical therapy; Combined therapy, ESWT with conventional physical therapy.


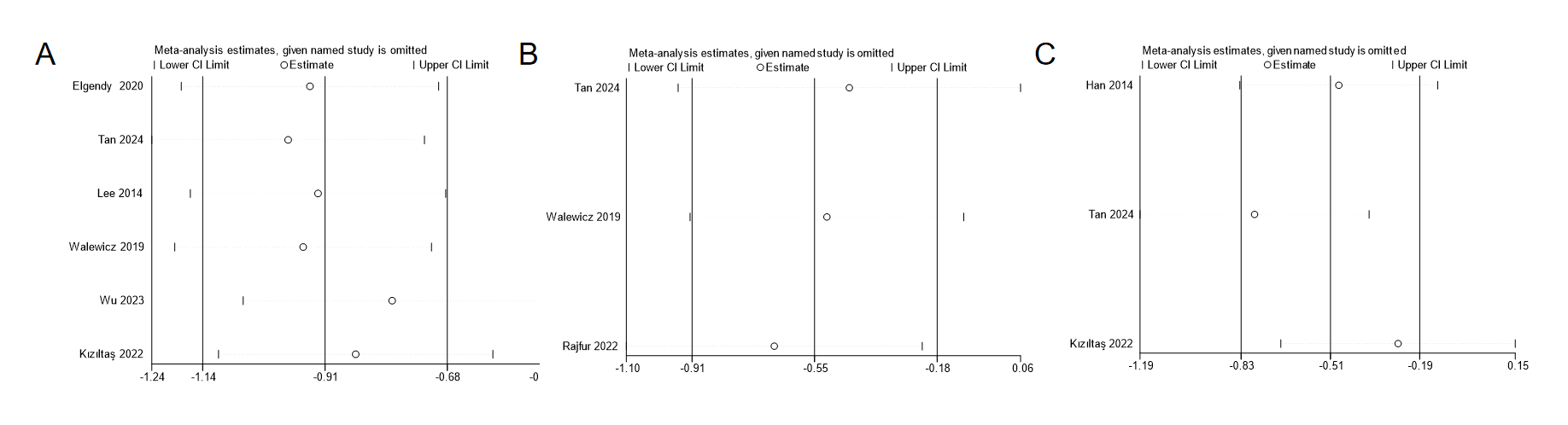


**Figure S7** Sensitivity analyses for pairwise comparisons. (A) Combined therapy compared with CPT in VAS; (B) Combined therapy compared with CPT in ODI; (C) ESWT compared with CPT in ODI. ESWT indicates extracorporeal shock wave therapy; ShamESWT, sham extracorporeal shock wave therapy; CPT, Conventional physical therapy; Combined therapy, ESWT with conventional physical therapy.

**Reference**

[1] Elgendy M, Mohamed M, Hussien H. Effect of extracorporeal shock wave on electromyographic activity of trunk muscles in non- specific chronic low back pain: a randomized controlled trial [J]. Eurasian Journal of Biosciences, 2020, 14: 6955-62. doi:

[2] Han H, Lee D, Lee S, et al. The effects of extracorporeal shock wave therapy on pain, disability, and depression of chronic low back pain patients [J]. J Phys Ther Sci, 2015, 27(2): 397-9. doi: 10.1589/jpts.27.397

[3] Guo X, Li L, Yan Z, et al. Efficacy and safety of treating chronic nonspecific low back pain with radial extracorporeal shock wave therapy (rESWT), rESWT combined with celecoxib and eperisone (C + E) or C + E alone: a prospective, randomized trial [J]. J Orthop Surg Res, 2021, 16(1): 705. doi: 10.1186/s13018-021-02848-x

[4] Tan K L, Wang R, Liu J J, et al. Effectiveness of focused extracorporeal shock wave versus manual therapy in postpartum patients with sacroiliac joint dysfunction: a prospective clinical trial [J]. J Orthop Surg Res, 2024, 19(1): 28. doi: 10.1186/s13018-023-04491-0

[5] Kızıltaş Ö, Okçu M, Tuncay F, et al. Comparison of the effectiveness of conventional physical therapy and extracorporeal shock wave therapy on pain, disability, functional status, and depression in patients with chronic low back pain [J]. Turk J Phys Med Rehabil, 2022, 68(3): 399-408. doi: 10.5606/tftrd.2022.8905

[6] Lee S, Lee D, Park J. Effects of extracorporeal shockwave therapy on patients with chronic low back pain and their dynamic balance ability [J]. J Phys Ther Sci, 2014, 26(1): 7-10. doi: 10.1589/jpts.26.7

[7] Rajfur K, Rajfur J, Matusz T, et al. Efficacy of Focused Extracorporeal Shock Wave Therapy in Chronic Low Back Pain: A Prospective Randomized 3-Month Follow-Up Study [J]. Med Sci Monit, 2022, 28: e936614. doi: 10.12659/msm.936614

[8] Moon Y E. Postoperative nausea and vomiting [J]. Korean J Anesthesiol, 2014, 67(3): 164-70. doi: 10.4097/kjae.2014.67.3.164

[9] Walewicz K, Taradaj J, Rajfur K, et al. The Effectiveness Of Radial Extracorporeal Shock Wave Therapy In Patients With Chronic Low Back Pain: A Prospective, Randomized, Single-Blinded Pilot Study [J]. Clin Interv Aging, 2019, 14: 1859-69. doi: 10.2147/cia.S224001

[10] Eftekharsadat B, Fasaie N, Golalizadeh D, et al. Comparison of efficacy of corticosteroid injection versus extracorporeal shock wave therapy on inferior trigger points in the quadratus lumborum muscle: a randomized clinical trial [J]. BMC Musculoskelet Disord, 2020, 21(1): 695. doi: 10.1186/s12891-020-03714-3

[11] Back C G N, Peron R, Lopes C V R, et al. Immediate effect of extracorporeal shockwave therapy in patients with chronic non-specific low back pain: A randomised placebo-controlled triple-blind trial [J]. Clin Rehabil, 2024, 38(8): 1080-90. doi: 10.1177/02692155241251844

[12] Fu Y S, Shih K S, Lin Y T, et al. Efficacy of ultrasound-guided piriformis muscle corticosteroid injection versus extracorporeal shockwave therapy in patients with piriformis syndrome: A randomized controlled trial [J]. J Formos Med Assoc, 2025. doi: 10.1016/j.jfma.2025.01.020

[13] Wu T, Wang D, Zhang X, et al. Comparison of pain relief and limb function improvement after extracorporeal shock wave therapy and thermomagnetic therapy in the treatment of low back pain [J]. Pak J Med Sci, 2023, 39(1): 268-73. doi: 10.12669/pjms.39.1.6668

[14] Nedelka T, Katolicky J, Nedelka J, et al. Efficacy of high-energy, focused ESWT in treatment of lumbar facet joint pain: a randomized sham-controlled trial [J]. Int J Surg, 2025, 111(7): 4177-86. doi: 10.1097/js9.0000000000002538
